# G protein-coupled receptor kinase 5 mediates Tazarotene-induced gene 1-induced growth suppression of human colon cancer cells

**DOI:** 10.1186/1471-2407-11-175

**Published:** 2011-05-17

**Authors:** Chang-Chieh Wu, Fu-Ming Tsai, Rong-Yaun Shyu, Ya-Ming Tsai, Chun-Hua Wang, Shun-Yuan Jiang

**Affiliations:** 1 Department of Surgery, Tri-Service General Hospital, 325 Chengung Rd, Sec 2, Taipei, 114 Taiwan; 2 Department of Research, Buddhist Tzu Chi General Hospital Taipei Branch, 289 Jianguo Rd, Sindian District, New Taipei City, 231 Taiwan; 3 Department of Internal Medicine, Buddhist Tzu Chi General Hospital Taipei Branch, 289 Jianguo Rd, Sindian District, New Taipei City, 231 Taiwan; 4 School of Medicine, Tzu Chi University, 701 Zhongyang Rd, Sec 3, Hualien, 970 Taiwan; 5 Department of Dermatology, Buddhist Tzu Chi General Hospital Taipei Branch, 289 Jianguo Rd, Sindian District, New Taipei City, 231 Taiwan

**Keywords:** RARRES1, TIG1, GRK5, microarray, colon cancer cells, tumour suppressor

## Abstract

**Background:**

Tazarotene-induced gene 1 (TIG1) is a retinoid-inducible type II tumour suppressor gene. The B isoform of TIG1 (TIG1B) inhibits growth and invasion of cancer cells. Expression of TIG1B is frequently downregulated in various cancer tissues; however, the expression and activities of the TIG1A isoform are yet to be reported. Therefore, this study investigated the effects of the TIG1A and TIG1B isoforms on cell growth and gene expression profiles using colon cancer cells.

**Methods:**

TIG1A and TIG1B stable clones derived from HCT116 and SW620 colon cancer cells were established using the GeneSwitch system; TIG1 isoform expression was induced by mifepristone treatment. Cell growth was assessed using the WST-1 cell proliferation and colony formation assays. RNA interference was used to examine the TIG1 mediating changes in cell growth. Gene expression profiles were determined using microarray and validated using real-time polymerase chain reaction, and Western blot analyses.

**Results:**

Both TIG1 isoforms were expressed at high levels in normal prostate and colon tissues and were downregulated in colon cancer cell lines. Both TIG1 isoforms significantly inhibited the growth of transiently transfected HCT116 cells and stably expressing TIG1A and TIG1B HCT116 and SW620 cells. Expression of 129 and 55 genes was altered upon induction of TIG1A and TIG1B expression, respectively, in stably expressing HCT116 cells. Of the genes analysed, 23 and 6 genes were upregulated and downregulated, respectively, in both TIG1A and TIG1B expressing cells. Upregulation of the G-protein-coupled receptor kinase 5 (GRK5) was confirmed using real-time polymerase chain reaction and Western blot analyses in both TIG1 stable cell lines. Silencing of TIG1A or GRK5 expression significantly decreased TIG1A-mediated cell growth suppression.

**Conclusions:**

Expression of both TIG1 isoforms was observed in normal prostate and colon tissues and was downregulated in colon cancer cell lines. Both TIG1 isoforms suppressed cell growth and stimulated GRK5 expression in HCT116 and SW620 cells. Knockdown of GRK5 expression alleviated TIG1A-induced growth suppression of HCT116 cells, suggesting that GRK5 mediates cell growth suppression by TIG1A. Thus, TIG1 may participate in the downregulation of G-protein coupled signaling by upregulating GRK5 expression.

## Background

The tazarotene-induced gene 1 (*TIG1*), also known as retinoic acid receptor responder 1 (*RARRES1*), is a retinoic acid receptor-responsive gene that was isolated from skin raft cultures treated with the synthetic retinoid, tazarotene [1]. The *TIG1 *gene, located on chromosome 3q25.32, is expressed in most normal tissues [2]. Loss of chromosome 3q heterozygocity has been reported in cancer tissues [3-5], while downregulation of TIG1 expression through promoter hypermethylation has been reported to occur in various carcinomas, including nasopharyngeal, esophageal, prostate, and colon [5-11].

The *TIG1 *gene encodes a transmembrane protein that contains a conserved latexin domain as well as a glycosylation signal and a hyaluronic acid-binding motif [1]. The protein shares 30% sequence similarity to the carboxypeptidase inhibitor, latexin, that regulates the size of the haematopoietic stem cell population [12]. However, it remains unknown whether TIG1 acts as a protease inhibitor. Expression of TIG1 is induced in differentiated psoriatic lesions and Caco-2 colon cancer cells treated with pro-differentiating agents, such as AGN1901683, a synthetic retinoid [1], or vitamin D3 [13]. In addition, TIG1 may regulate cellular differentiation. Indeed, the expression of TIG1 is closely associated with the differentiation of colorectal adenocarcinoma [14] and mesenchymal stem cells derived from adipocytes [15]. In the prostate, TIG1 expression is progressively lost from benign tissues to malignant lesions, while the ectopic expression of TIG1 suppresses cellular growth *in vitro *[7,10] and *in vivo *[2]. Further, TIG1 has been reported to be differentially expressed in spontaneously regressing melanomas in a MeLiM swine model of melanoma [16]. Finally, TIG1 expression suppresses the invasion of prostate cancer cells [2]. Conversely, knock-down of TIG1 expression increases invasion of Epstein-Barr virus-infected nasopharyngeal carcinoma cells [7]. Thus, TIG1 is a tumour suppressor that prevents carcinogenesis in several tissue types. However, the molecular mechanisms underlying the activities of TIG1 on cell growth, invasion and differentiation have not been reported.

According to information obtained from AceView, there are 8 putative protein isoforms encoded by the *TIG1 *gene [17]. Studies analysing the suppression of cell growth and invasion by TIG1 have focused on the TIG1B isoform [GenBank:NP_002879.2] that comprises 228 amino acids [2,7,10]; however, characterisation of the expression and activity of the TIG1A isoform is yet to be reported. The TIG1A isoform [GenBank:NP_996846.1] contains 294 amino acids and shares the same N-terminal 224 amino acid sequence as TIG1B. In this study, we investigated the effects of both TIG1 isoforms on growth and gene expression profiles in HCT116 colon cancer cells to test the hypothesis that TIG1A also suppresses colon cell growth. To determine the proteins associated with TIG1A- and TIG1B-related growth of colon cancer cells, inducible stable cells that expressed TIG1A or TIG1B protein isoforms in HCT116 and SW620 colon cancer cells were established using the GeneSwitch™ system [18]. In the GeneSwitch™ system, a hybrid regulatory protein containing a DNA binding domain from the yeast GAL4 protein, a truncated ligand binding domain from the human progesterone receptor and an activation domain from the human NF-kB protein is expressed in the pSwitch plasmid. The system allows the induction of TIG1A and TIG1B expression in cells harboring the hybrid regulatory protein upon exposure to the synthetic progestin, mifepristone (MFP). Gene expression profile changes upon TIG1A and TIG1B expression were analysed by microarray. G-protein-coupled receptor kinase 5 (GRK5), a serine/threonine kinase, which suppresses the activation of ligand-activated G-protein-coupled receptors (GPCRs) [19], was upregulated by both TIG1 isoforms. The importance of GRK5 on TIG1-induced growth suppression was analysed using RNA interference.

## Methods

### Construction of expression vectors

Constitutive expression vectors encoding myc and his-tagged TIG1A (pTIG1A-myc) and TIG1B (pTIG1B-myc) fusion proteins were constructed. The TIG1A and TIG1B cDNA fragments were amplified from human SC-M1 gastric cancer cells, obtained from Dr. C.-L. Meng (National Defense Medical Center, Taipei, Taiwan), using TIG1A-specific primers (sense, 5'-CTCTGAATTCCTGCGTCCATGCAGCCC-3' and antisense, 5'-GCGCGGATCCGAAATTACTAAGCTCTG-3') and TIG1B-specific primers (sense, 5'-CTCTGAATTCCTGCGTCCATGCAGCCC-3' and antisense, 5'-CGGACGGATCCGGTTTTTCTTACCCAC-3'). Amplified cDNA fragments were digested with *Eco*RI and *Bam*HI, and then subcloned in-frame into the multicloning site of the pcDNA3.1-myc-his A expression vector (Invitrogen, Carlsbad, CA, USA). To generate inducible TIG1A and TIG1B expression plasmids (pTIG1A-V5 and pTIG1B-V5, respectively), *Hin*dIII- and *Bam*HI-digested TIG1 cDNA fragments isolated from pTIG1A-myc or pTIG1B-myc were cloned in-frame into the pGene-V5-his B vector (Invitrogen).

### Cell culture and transfection

HCT116 colon cancer cells were maintained in growth medium consisting of McCoy's 5A medium (BioWest, Nuaille, France) supplemented with 25 mM HEPES, 26 mM NaHCO_3_, 2 mM L-glutamine, 100 units/mL penicillin, 100 μg/mL streptomycin, and 10% fetal bovine serum (FBS; Hyclone, Logan, Utah, USA) at 37°C and 5% CO_2_. SW620 cells were maintained in growth medium consisting of Leibovitz's L15 medium (GIBCO BRL, Grand island, NY, USA) supplemented with 25 mM HEPES, 26 mM NaHCO3, 2 mM L-glutamine, 100 units/mL penicillin, 100 μg/mL streptomycin, and 10% FBS at 37°C without CO_2_. HT-29, LS174T, and SW480 colon cells were maintained in Dulbecco's Modified Essential Medium (GIBCO BRL) supplemented with 10% FBS. CC-M1, CC-M2, CC-M3, DLD-1, and COLO205 colon cells were maintained in RPMI medium (GIBCO BRL) supplemented with 25 mM HEPES, 26 mM NaHCO_3_, 2 mM L-glutamine, 100 units/mL penicillin, 100 μg/mL streptomycin, and 10% FBS at 37°C and 5% CO_2_. Colon cancer cells were obtained from Food Industry Research and Development Institute (Sinju, Taiwan).

HCT116 cells plated in 6-cm culture dishes were transfected with expression vectors using liposome-mediated transfection. Briefly, plasmids or small interfering RNAs (siRNAs) and lipofectamine (Invitrogen) were diluted in Opti-MEM medium (GIBCO BRL) and then mixed at room temperature for 15 min. The nucleic acid-lipofectamine complexes were then added to cells for 2.5 h at 37°C. Cells were then refreshed with complete medium for 24 h at 37°C for further analysis.

### Establishment of inducible TIG1 stable clones

Inducible TIG1 stable clones were established using the GeneSwitch system (Invitrogen) as previously described with minor modifications [20]. Briefly, HCT116 cells plated in 10-cm dishes were first transfected with the pSwitch expression vector (Invitrogen) that expressed the hybrid regulatory protein using lipofectamine, and then selected in hygromycin (400 μg/mL, Invitrogen)-containing medium. Stable HCT116/Switch clones were selected by limiting dilution and validated by analysing the induced expression of β-galactosidase followed by the transfection with pLacZ/V5 (Invitrogen) and then incubation with 10 nM MFP. HCT116/Switch cells that expressed the MFP-dependent transactivator were selected for subsequent transfection with pTIG1A-V5, pTIG1B-V5, or the control vector (pGene-V5-his B). Stable cells were selected in medium containing hygromycin and zeocin (125 μg/mL, Invitrogen). Inducible expression of TIG1A and TIG1B was validated by immunocytochemistry and Western blot analyses. Stable clones were maintained in McCoy's 5A growth medium supplemented with hygromycin and zeocin. Cells used for further experiments were plated in medium without hygromycin and zeocin. Representative inducible TIG1A (clone 2), TIG1B (clone 8), and control (2G5) stable clones were selected for analysis.

Using similar methods, inducible TIG1A (clone 13), TIG1B (clone 7), and control (clone 1) stable clones were established in SW620 colon cancer cells. Stable cells were maintained in L15 growth medium supplemented with 800 μg/mL hygromycin and 400 μg/mL zeocin. Cells were monitored monthly for mycoplasma contamination using the DNA intercalating dye, 4'6-diamidino-2-phenylindole (Sigma, St. Louis, MO, USA).

### Analysis of cellular proliferation

HCT116 cells were seeded at 2 × 10^4 ^cells per well in 24-well plates overnight and then transfected with constitutive TIG1A or TIG1B expression vector or control vector for 48 h. WST-1 reagent (100 μL Roche Diagnostics, Mannheim, Germany) was then added to the cells for 4 h at 37°C. Absorbance at 450 and 650 nm was assessed. The percentage of proliferative TIG1A or TIG1B transfected cells relative to controls was defined as [(A_450_-A_650_) of TIG1A or TIG1B transfected cells/(A_450_-A_650_) of control transfected cells] × 100%. Alternatively, control or TIG1 stable clones were plated overnight in growth medium free of hygromycin and zeocin. The cells were then incubated in growth medium containing MFP (0.1 - 100 nM) or ethanol (0.1%) vehicle for 48 h. The percentage of MFP-treated proliferative cells relative to controls was defined as [(A_450_-A_650_) of MFP-treated cells/(A_450_-A_650_) of ethanol-treated cells] × 100%. All experiments were performed in triplicate.

### Colony formation assay

HCT116 cells were plated in 6-well dishes and transfected with pTIG1A-myc, pTIG1B-myc expression vector, or control vector for 3 h after which they were incubated in growth medium for 1 day. Cells were then cultured in medium containing 1000 μg/mL G-418 for 8 days with medium changes performed every other day. The number of colonies was determined using crystal violet staining as described previously [21]. All experiments were performed in triplicate.

### Microarray analysis

Inducible TIG1A, TIG1B, and control stable HCT116 cell clones plated overnight in 10-cm dishes in growth medium were stimulated with growth medium containing 5 nM MFP for 24 h. Cells were washed twice with ice-cold PBS (3.2 mM Na_2_HPO_4_, 0.5 mM KH_2_PO_4_, 1.3 mM KCl, 135 mM NaCl, pH 7.4), detached by scraping and isolated by centrifugation at 1500 × g for 5 min at 4°C. Three independent samples were collected for microarray analysis.

Total RNA was extracted using the RNeasy system (Qiagen, Valencia, CA, USA). The integrity of the RNA was assessed using a 2100 Bioanalyzer (Agilent Technologies, Palo Alto, CA, USA). Integrity values exceeded 9.7 in all tested samples. Affymetrix microarray analysis was carried out according to the manufacturer's protocol (Affymetrix, Santa Clara, CA, USA). Briefly, cRNA probes were prepared from 10 μg of total RNA using an Affymetrix One Cycle cDNA synthesis kit (Affymetrix). The biotinylated cRNA was subsequently fragmented and hybridised to each array at 45°C for over 16 h. cRNA was hybridised to the HGU133-2 plus whole genome GeneChip containing 54,675 transcripts. Quality assessment was performed using the GeneChip Operating System 1.1.1 (Affymetrix) using global scaling to a target signal of 500. Median background was 38.0 - 40.7. Median scaling factors were 6.5 - 9.2, and noise values ranged from 1.6 - 1.8. High overall array and RNA quality were confirmed.

### Normalisation and statistical analysis

Gene expression data underwent secondary analysis using the GeneSpring GX program (Agilent Technologies, Santa Clara, CA, USA). Briefly, gene expression data from the microarray chips were rescaled (after preliminary analysis) for a direct comparison between all chips. Data were filtered based on both Detection Call and Signal Log Ratio for comparisons between groups (control and TIG1A or control and TIG1B). Data declared "Absent" in all samples were filtered out. Samples had to be declared "Present" in at least 2 of 3 chips within a group to be maintained within the set. The log base 2.0 of the fold change was used as an additional filter. Filtered genes identified to be differentially expressed by 2-fold or greater in 2 of 3 chips were analysed for functional gene clusters using GeneSpring. Statistical analyses were performed using one-way ANOVA (*P *< 0.05) and *t*-tests. The program was used to determine functional clusters by statistical representation of individual genes in specific categories relative to all genes in the same category on the array. Signal pathways of differentially expressed genes were validated using the Protein Analysis Through Evolution Relationships (PANTHER™) protein classification system (Applied Biosystems, Foster City, CA, USA). Original microarray data have been deposited in the Gene Expression Omnibus data bank (accession number: GSE23882).

### RNA isolation and quantitative real-time reverse transcription polymerase chain reaction (RT-PCR)

Total RNA was extracted using TRIZOL reagent (Invitrogen). cDNA was prepared by incubating a 20 μL mixture containing 5 μg total RNA, 1 unit Moloney murine leukemia virus (MuLV) reverse transcriptase (Invitrogen), 0.5 μg oligo-dT_12-18 _in 50 mM Tris-HCl, pH 8.3, 75 mM KCl, 4.25 mM MgCl_2_, 0.5 mM dNTP, and 1 unit RNaseout recombinant RNase inhibitor (Invitrogen) at 37°C for 1 h. Real-time RT-PCR was performed using the ABI prism 7900HT sequencer detector system (Applied Biosystems) and Qiagen's Quantitect SYBR Green PCR kit (Qiagen) following the instructions provided. In brief, reaction mixtures (25 μl of total volume) containing 250 ng cDNA, gene-specific forward and reverse primers (1 μM), and 12.5 μL of 2× Quantitect SYBR Green PCR Master mix were cycled as follows: 1 cycle at 95°C for 10 min; 40 cycles at 95°C for 15 seconds; and 60°C for 1 min. Negative controls without the template were run in parallel to assess the overall specificity of the reaction. PCR primers used for amplification included the following: beta-actin (sense, 5'-TCCCTGGAGAAGAGCTACG-3' and antisense, 5'-GTAGTTTCGTGGATGCCACA-3'), TIG1 (sense, 5'-CGTGGTCTTCAGCACAGAGCG-3' and antisense, 5'-CCCAAACGTCCCTCACCTTCC-3'), GRK5 (sense, 5'-GACCACACAGACGACGACTTC-3' and antisense, 5'-CGTTCAGCTCCTTAAAGCATTC-3'), TIG1A (sense, 5'- CCGAGCCAACTTTCCTGCGTCC-3' and antisense, 5'-CCCTGAGGAACCTGCTGGTGACTG-3'), TIG1B (sense, 5'-CGAGCCAACTTTCCTGCGTC-3' and antisense, 5'-CTTGTGGCACAAGTTAATTTTCAGG-3'), and TCF7 (sense, 5'-TCCAGAGCCCCTGGAGGACG-3' and antisense, 5'-GGGCTGATTGGCCTTGTGCA-3'). Relative TIG1A, TIG1B, TCF7, or GRK5 mRNA levels in MFP-induced stable cells after normalisation to the vehicle treated control cells were calculated as follows: 2^- ΔΔ^C_T _= 2/([C_T _of TIG1A in MFP-treated cells]- [C_T _of actin in MFP-treated cells])- ([C_T _of TIG1A in control cells]- [C_T _of actin in control cells]). All experiments were performed in triplicate.

Relative expression of TIG1A and TIG1B in normal tissues and colon cancer cell lines was determined using the icycler system (Bio-Rad Laboratories, Hercules, CA, USA). Total RNAs from normal tissues obtained from Clontech Laboratories (Clontech, Palo Alto, CA, USA) and colon cancer cells purified as described above were reverse transcribed. Real time RT-PCR was performed in reaction mixtures (20 μL total volume) containing 100 ng cDNA, gene-specific forward and reverse primers (160 nM), and 10 μL 2× iQ™ SYBR^® ^Green Supermix (Bio-Rad). The real-time cycler conditions were as follows: 1 cycle at 94°C for 3 min and 40 cycles at 94°C for 30 seconds, 61°C for 30 seconds, and 72°C for 30 seconds. Primers used for amplification were as follows: TIG1A (sense, 5'-CCTGTCGCATTCACTTGGTCTG-3' and antisense, 5'-CGGAGG CTTCTTCTGGTGTCTG-3'), TIG1B (sense, 5'-TCTGAGACTCATCTGGGATTTG-3' and antisense, 5'-CTTTGATTGTAACTCTTGTGGC-3'), and glyceraldehyde 3-phosphate dehydrogenase (GAPDH; sense, 5'-TCCATGCCATCACT GCCACCC-3' and antisense, 5'-GACGCCTGCTTCACCACCTTC-3'). Relative TIG1A or TIG1B mRNA levels were calculated using the following equation: 10,000 × 2^-ΔΔ^C_T _= 10,000 × 2/([C_T _of TIG1 in tissue A]- [C_T _of GAPDH in tissue A])- ([C_T _of TIG1 in colon]- [C_T _of GAPDH in colon]). All experiments were performed in triplicate.

### Western blot analysis

Total cytosol extracts were prepared in mild lysis buffer (25 mM HEPES, pH 7.5, 150 mM NaCl, 1% Igepal CA-630, 10 mM MgCl_2_, 1 mM EDTA, and 10% glycerol) containing protease inhibitors (20 μg/mL aprotinin and 20 μg/mL phenylmethylsulfonyl fluoride) and phosphatase inhibitors (2 mM NaF and 1 mM Na_3_VO_4_). Proteins (5 to 100 μg) were separated using 10-12% polyacrylamide gels containing sodium dodecyl sulfate and were then transferred to polyvinylidene difluoride membranes. After blocking, membranes were incubated with anti-V5 (Invitrogen), anti-myc (Invitrogen), anti-GRK5 (Santa Cruz Biotechnology, Santa Cruz, CA, USA), or anti-actin (Sigma) antibody for 12 h at 4°C. Membranes were then incubated with horseradish peroxidase-conjugated goat anti-mouse or anti-rabbit antibodies at room temperature for 1 h. Specific protein bands were developed using Amersham ECL (Amersham, Bucks, UK). Relative protein expression levels were quantified by normalising to actin expression levels.

### RNA interference

siRNA oligonucleotides were synthesised by Ambion (Austin, TX, USA). Three TIG1 siRNAs targeted to nucleotides 488 to 508 (5'-GUACAAGUUACAUUGAUGGtt-3'), 540 to 560 (5'-GUUGUAAAGCAGGUAAUCCtc-3'), and 596 to 616 (5'-CCAUGAUUAUCAGGUAUGCtg-3') were synthesised based on Genbank accession NM_26963. The TIG1 siRNAs are specific for both TIG1A and TIG1B isoforms. GRK5 siRNAs targeted to nucleotides 452 to 472 (5'-UAACACUCCAGCCCAGGCCtg-3'), 2158 to 2178 (5'-GAAAUUUCUUUGAGAAACCtc-3'), and 2406 to 2426 (5'-GCACAGAAAAUAUACACCCtc-3') were synthesised according to Genbank accession NM_005308. The Silencer^® ^negative control#1 siRNA (Ambion) was used as a negative control. Inducible TIG1 or control stable cells were cultured for 24 h in 6-well plates and then transfected with 30 nM siRNAs using Lipofectamine according to the instructions provided. After transfection, cells were incubated in the presence or absence of MFP for 48 h, and then analysed for growth.

### Statistical analysis

Numerical data are shown as mean ± standard deviation (SD) of triplicates from each independent biological sample. Cell proliferation, colony formation, and real-time RT-PCR data were analysed using student's *t *test. *P*-values less than 0.05 were considered to be statistically significant. Microarray data were analysed by one-way ANOVA using GeneSpring GX software.

## Results

### Expression profiles of both TIG1 isoforms in normal tissues and colon cancer cell lines

**Figure 1:**
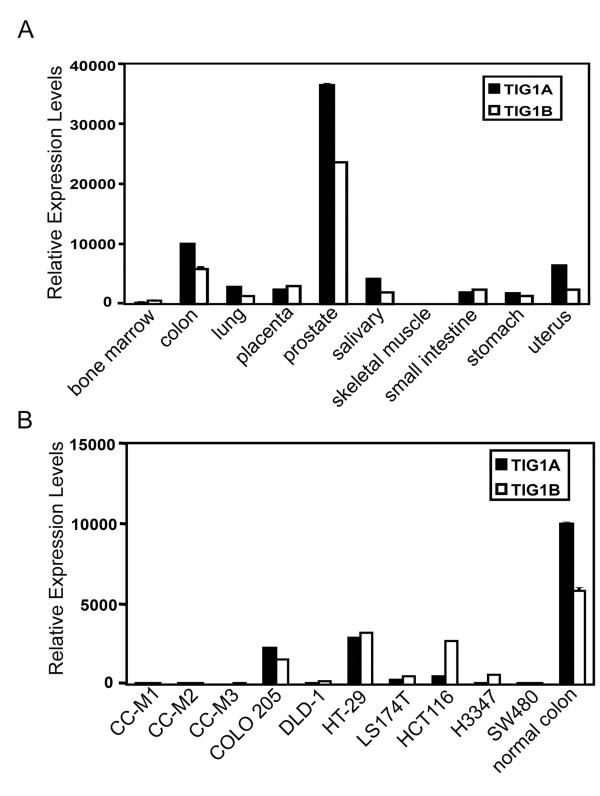
**TIG1A and TIG1B expression in normal tissues and colon cancer cell lines**. Real time RT-PCR analysis of TIG1A and TIG1B mRNAs from normal human tissues (A) and colon cancer cell lines (B). Relative TIG1A and TIG1B mRNA levels were first normalised to GAPDH levels and then normalised to TIG1A levels in the normal colon, which was set at 10,000 units. Data represent the means and SDs from triplicate sample wells.

Relative expression levels of TIG1A and TIG1B were evaluated in normal human tissues and in colon cancer cell lines using real-time RT-PCR. Both TIG1A and TIG1B isoforms were expressed at high levels in the prostate and colon. However, expression was barely detectable in bone marrow and skeletal muscle (Figure 1A). Compared with normal colon tissue, expression of both TIG1 isoforms was decreased in all colon cancer cell lines analysed (Figure 1B). Seven of the cell lines analysed expressed less than 2 and 11% of normal TIG1A and TIG1B expression levels, respectively.

### Effect of TIG1 isoforms on growth of HCT116 cells

**Figure 2:**
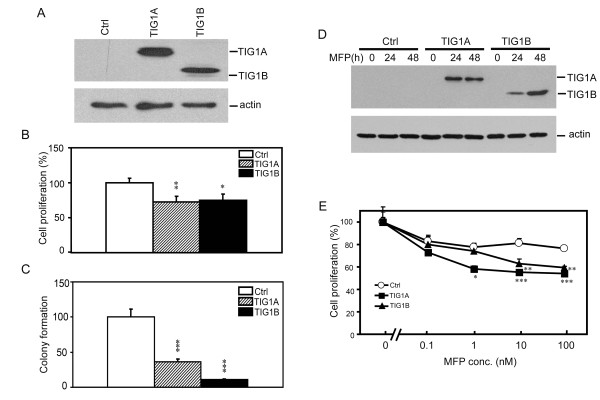
**Suppression of HCT116 cell growth by TIG1A and TIG1B**. (A) Western blot analysis of TIG1 isoforms in transiently transfected HCT116 cells. Cells were transiently transfected with the indicated expression vectors for 24 h. Expression of TIG1A and TIG1B was detected using an anti-myc antibody. β-actin expression served as a loading control. (B) Analysis of the effects of TIG1 isoforms on cell proliferation using the WST-1 cell proliferation assay. Cells were transiently transfected with the indicated constitutive expression vectors for 48 h after which cell proliferation was measured. Data represent the mean and SD of the percentage of cell proliferation performed in triplicate and are representative of two independent experiments. (C) The effects of TIG1 isoforms on HCT116 colony formation. Cells were transiently transfected with the indicated constitutive expression vector, and colony formation was determined. Data represent the mean and SD of percentage colony formation normalised to the control performed in triplicate. Results are representative of three independent experiments. (D) Analysis of TIG1A and TIG1B expression in stable cell lines. TIG1A, TIG1B, or control stable clones were incubated in the presence or absence of 5 nM MFP for 0 - 48 h, and expression of TIG1 isoforms was analysed using Western blots analysis and anti-V5 antibody. β-actin expression served as a loading control. (E) Effects of TIG1 isoform expression on the growth of TIG1 stable cells. TIG1 and control stable clones were incubated with various concentrations of MFP or vehicle alone for 48 h. Cell proliferation was measured using the WST-1 assay. Data represent the mean and SD of percentage of cell proliferation performed in triplicate. Results are representative of three independent experiments. *, *P *< 0.05; **, *P *< 0.01; ***, *P *< 0.001 compared with control stable cells incubated with the indicated concentration of MFP. Ctrl, control.

The activities of TIG1A and TIG1B were measured in HCT116 cells that exhibited high transfection efficiency and were previously shown to have a hypermethylated *TIG1 *gene. Ectopically expressed recombinant TIG1A and TIG1B protein isoforms with the expected molecular weights of 36.7 and 29.2 kDa, respectively, were detected in HCT116 cells after transient transfection for 24 h (Figure 2A). The effects of TIG1A and TIG1B expression on HCT116 cell growth were first analysed using the WST-1 cell proliferation assay. After 48 h, HCT116 cell growth was significantly inhibited by 27 and 25% following transient transfection with constitutive TIG1A and TIG1B expression vectors, respectively (Figure 2B). Similarly, colony formation of HCT116 cells was significantly suppressed by 63.3 and 89.2% upon transient transfection with TIG1A and TIG1B expression vectors, respectively and maintenance in G418-containing media for 8 days (Figure 2C). The effect of TIG1 isoform expression on cell death was also determined by measuring the release of lactate dehydrogenase in transiently transfected HCT116 cells. Significantly increased cell death (10.3 to 24.6%) was detected in TIG1B-transfected cells; however, cell death was weakly, but significantly, repressed by 5.3 to 12.4% in TIG1A-transfected cells (Additional file 1).

As production of stable cells constitutively expressing TIG1A or TIG1B protein was unsuccessful, inducible TIG1A, TIG1B, and control stable cells were established from HCT116 cells using the GeneSwitch system [18] in which target protein expression is induced by exposure to MFP. Exposure to 5 nM for 24 to 48 h profoundly increased expression of TIG1A or TIG1B in respective TIG1A or TIG1B stable cells, but not the control stable cells (Figure 2D). Expression of both TIG1A and TIG1B was induced after exposure to 0.1 - 100 nM MFP for 48 h (data not shown).

To verify that TIG1A and TIG1B expression influenced HCT116 cell growth, proliferation was assessed in stable clones incubated with various concentrations of MFP (Figure 2E). Cell proliferation, as determined by WST-1 cell proliferation assay, was inhibited by MFP (0.1 - 100 nM) in control stable cells with a maximal inhibition of 25.4% by 100 nM MFP. Induction of TIG1A and TIG1B expression by MFP treatment resulted in a concentration-dependent inhibition of cell growth in both TIG1A and TIG1B stable cells. When compared with MFP-treated control stable cells, 29.6 and 22.2% inhibition of cell growth was observed in TIG1A and TIG1B cells, respectively, incubated with 100 nM MFP for 48 h (Figure 2E). The release of lactate dehydrogenase as an indicator of cell cytotoxicity in stable cells exposed to 5 nM MFP for 1 to 2 days was also analysed; however, no increase in cell death was observed (data not shown). As 5 nM MFP efficiently induced expression of recombinant TIG1 isoforms, this concentration was chosen for microarray analysis to avoid the identification of genes differentially regulated by TIG1A or TIG1B in cells exposed to a high concentration of MFP.

### Analysis of gene expression profiles in TIG1A and TIG1B stable cells

**Figure 3:**
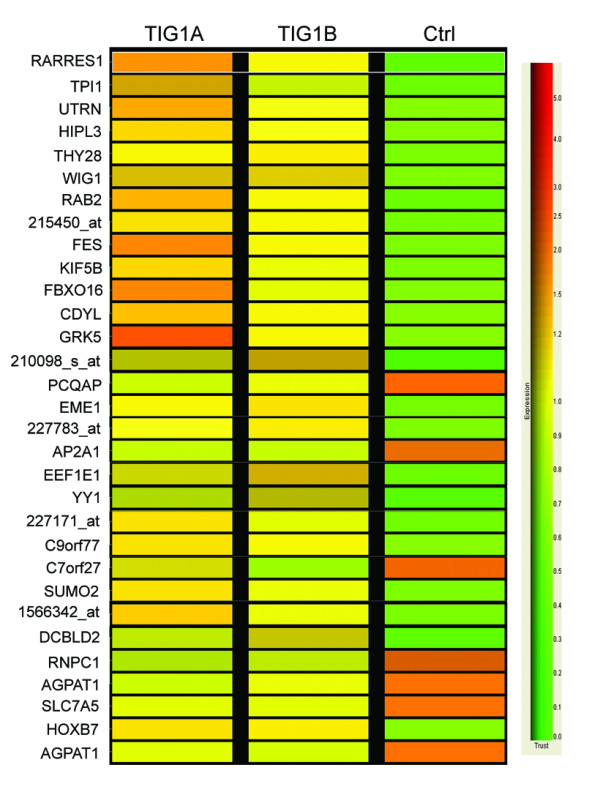
**Microarray analysis of genes differentially regulated by both TIG1A and TIG1B in HCT116 cells**. Control (Ctrl), TIG1A, and TIG1B stable cells were treated with 5 nM MFP for 24 h. Profiles of gene expression were then determined using microarray analysis. After comparing to the gene expression in control cells, genes upregulated or downregulated by greater than two-fold in both TIG1A and TIG1B stable cells were subjected to hierarchical clustering. Average expression was defined by the GeneSpring^® ^software. The relative scale of upregulation (red) or downregulation (green) of gene expression is shown in the right panel.

To examine the effects of TIG1A and TIG1B on the gene expression profile of HCT116 cells, stable cells were incubated with 5 nM MFP for 24 h after which they were analysed using the human HGU133 Plus 2.0 microarray (Additional file 2). Genes differentially expressed by greater than two-fold (relative to expression in control cells) in TIG1A (Additional file 3) or TIG1B (Additional file 4) stable cells were selected for analysis. Following induction of TIG1A expression, a total of 108 and 21 genes were significantly upregulated or downregulated, respectively (Additional file 3). Similarly, upon induction of TIG1B expression, a total of 41 and 14 genes were upregulated or downregulated, respectively (Additional file 4). A total of 23 of the upregulated genes and 6 of downregulated genes identified were detected upon both TIG1A and TIG1B induction (Figure 3, Additional file 5). Among the 29 genes regulated by both TIG1A and TIG1B isoforms, genes related to the p53 (wild type p53-induced gene 1 [WIG1] and small ubiquitin-like modifier 2 [SUMO2]), Wnt (homeobox B7 [HOXB7]), interleukin (eukaryotic translation elongation factor 1 epsilon 1 [EEF1E1]), chemokine and cytokine (GRK5 and EEF1E1), and G-protein coupled receptor (GCPR; GRK5) signaling pathways were identified.

### Upregulation of GRK5 and TCF7 upon TIG1A and TIG1B expression in HCT116 cells

**Figure 4:**
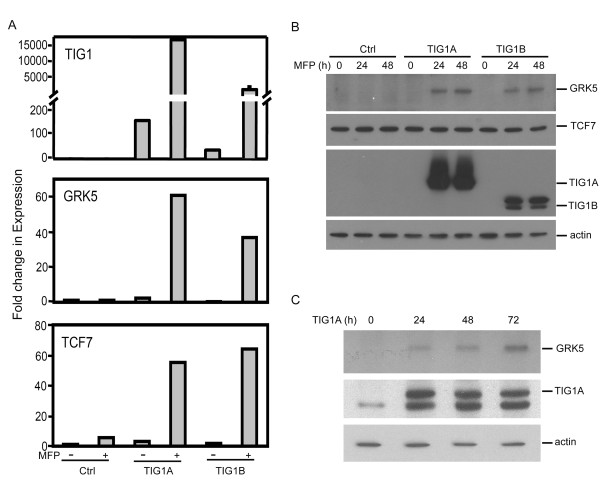
**Effects of TIG1A and TIG1B on GRK5 and TCF7 expression in HCT116 cells**. (A) Real time RT-PCR analysis of gene expression in inducible stable cells. Stable HCT116 clones were treated with 5 nM MFP for 24 h. Total RNA was extracted and relative levels of the indicated mRNAs were measured by real-time RT-PCR. Data represent the mean and SD of fold changes in gene expression performed in triplicate after normalisation to the results obtained from vehicle-treated control stable cells. Results shown are representative of 2 independent experiments. (B) Western blot analysis of the effects of TIG1 isoforms on GRK5 and TCF7 expression in stable cells. Stable HCT116 clones were treated with 5 nM MFP for 0 to 48 h. Cell lysates were prepared, and expression of TIG1A, TIG1B, GRK5, TCF7 and β-actin was determined by Western blot analysis. (C) Up-regulation of GRK5 expression in HCT116 cells transiently transfected with a TIG1A expression plasmid. Cells were transiently transfected with constitutive TIG1A expression vector for 0 to 72 h. TIG1A, GRK5 and β-actin expression from total cellular lysates was determined by Western blot analysis. Ctrl, control.

The Wnt signaling pathway is stimulated by the GPCR ligand, prostaglandin E2, through enhanced nuclear β-catenin localisation and β-catenin/TCF-mediated transactivation [22]. GRK5 is a member of family proteins that attenuate GPCR activities. Therefore, GRK5 and TCF7 were selected for further analysis. Upon exposure to 5 nM MFP for 24 h, TIG1A and TIG1B mRNAs levels were increased by 107.1- and 29.2-fold, respectively (Figure 4A). Compared with control stable cells, basal TIG1A and TIG1B mRNA levels in TIG1A and TIG1B stable cells not exposed to MFP were 166.2- and 37.8-fold higher, respectively. These results suggest the presence of a weak basal activity of the inducible promoter although basal levels of TIG1 fusion proteins were not detected by Western blot analysis (Figure 2D and Figure 4B). Induced expression of TIG1A and TIG1B by MFP treatment resulted in significantly increased GRK5 (24.3- and 45.9-fold, respectively) and TCF7 (16.3- and 35.8-fold, respectively) mRNA levels (Figure 4A). Basal levels of GRK5 and TCF7 mRNA in TIG1A and TIG1B stable cells were similar to those in control cells.

TIG1-mediated regulation of GRK5 and TCF7 protein expression was examined using Western blot analysis. No GRK5 protein was detected in MFP-treated control cells or in TIG1A and TIG1B stable cells in the absence of MFP treatment. Increased GRK5 protein expression (65 kDa) was observed in TIG1A and TIG1B stable cells exposed to 5 nM MFP for 24 to 48 h (Figure 4B). Compared with cells exposed to MFP for 24 h, GRK5 protein levels were increased by 22 and 75% in TIG1A and TIG1B stable cells exposed to MFP for 48 h, respectively. Similarly, basal GRK5 protein was not detected in HCT116 cells (Figure 4C), while GRK5 protein expression was upregulated in HCT116 cells transiently transfected with the constitutive TIG1A expression vector for 24 to 72 h. GRK5 protein levels were increased (relative to 24 h transfected cell levels) by 58 and 179% after transfection for 48 and 72 h, respectively. Regardless of TCF7 mRNA upregulation, neither TIG1A nor TIG1B expression influenced TCF7 protein levels after exposure to MFP for 24 and 48 h (Figure 4B).

### Silencing of GRK5 expression decreases TIG1-mediated suppression of cell growth

**Figure 5:**
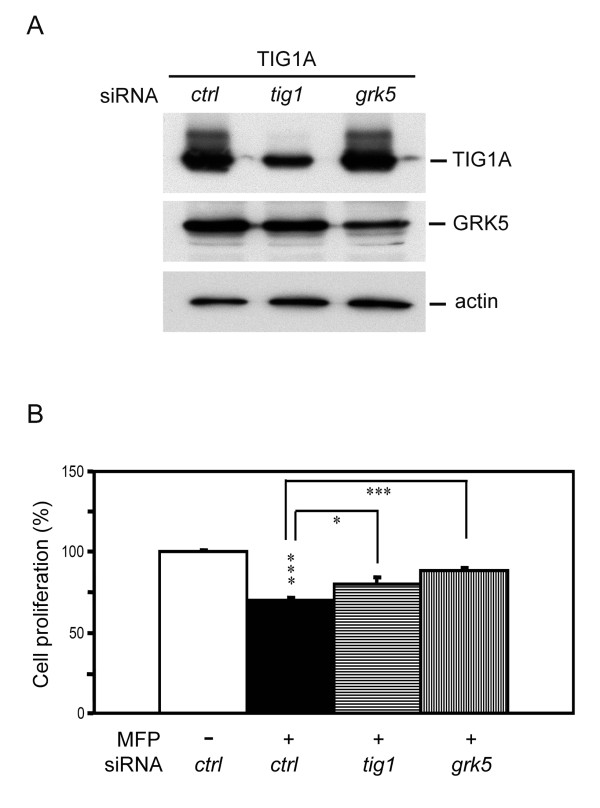
**Effects of TIG1A and GRK5 siRNAs on TIG1A-mediated suppression of HCT116 cell growth**. (A) TIG1A cells were transfected with the indicated siRNA after which they were immediately incubated with MFP (5 nM) for 24 h. Expression of TIG1A, GRK5, and actin was examined by Western blot analysis using anti-myc, anti-GRK5, and anti-actin antibodies, respectively. (B) Inducible TIG1A stable cells were plated in triplicate overnight, and then transfected with the indicated siRNA, and incubated in the presence or absence of 5 nM MFP for 48 h. Cell proliferation was measured using the WST-1 method. Relative levels of cell growth were normalised to that of control siRNA transfected cells without MFP treatment. *, *P *< 0.05 and ***, *P *< 0.001.

To evaluate the role of GRK5 in TIG1-mediated inhibition of HCT116 cell proliferation, RNA interference was employed. TIG1A stable cells were transiently transfected with siRNAs specific for both TIG1 isoforms or GRK5 and then immediately exposed to 5 nM MFP for 24 h. TIG1A protein levels were reduced by 53.7% in cells transfected with TIG1 siRNA, while GRK5 protein levels was reduced by 51.7% in GRK5 siRNA transfected cells, determined by Western blotting analysis (Figure 5A). In control siRNA transfected TIG1A cells, cellular growth was significantly inhibited to 70.2% of control levels upon MFP exposure that induced TIG1A expression. Silencing of TIG1A or GRK5 expression significantly alleviated TIG1A-mediated growth suppression to 80 and 88.2% of control growth, respectively (*P *< 0.05 and *P *< 0.001, respectively; Figure 5B). Levels of cell growth in MFP-treated TIG1A cells transfected with GRK5 siRNA were slightly higher, but not significant, than that of cells transfected with TIG1 siRNA.

### Both TIG1A and TIG1B isoforms suppressed cell growth and induced GRK5 expression in SW620 colon cancer cells

**Figure 6:**
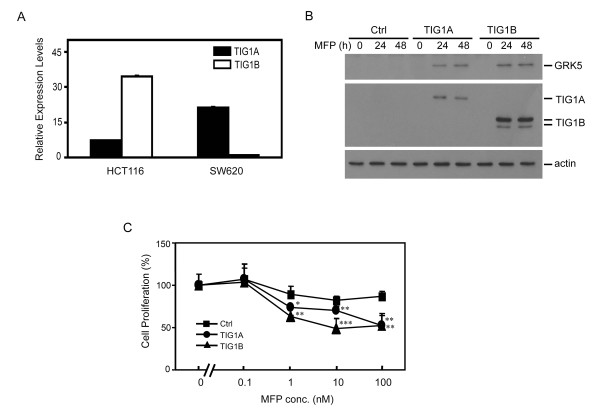
**Effect of stable TIG1 expression on GRK5 expression and cell growth of SW620 colon cancer cells**. (A) Real time RT-PCR analysis of TIG1A and TIG1B mRNA levels from HCT116 and SW620 colon cancer cell lines. (B) GRK5 expression in inducible TIG1 stable cells established from SW620 cells. Control or inducible TIG1A and TIG1B stable cells were plated overnight and then incubated with 5 nM MFP for 0 to 48 h. Expression of GRK5, TIG1 isoforms, and actin were analysed by Western blotting. (C) Effects of TIG1 isoform expression on SW620 cell growth. Inducible TIG1 and control stable clones were plated in triplicate overnight and then incubated with the indicated concentrations of MFP for 48 h. Cell proliferation was measured using the WST-1 method. Data represent the mean and SD of the percentage of cell proliferation performed in triplicate. *, *P *< 0.05; **, *P *< 0.01; ***, *P *< 0.001 compared with control stable cells incubated with the indicated concentration of MFP. Ctrl, control.

To verify the cell independent effect of TIG1 isoform expression in colon cancer cells and to evaluate the effect of TIG1 on cells established from metastatic lesions, TIGA, TIG1B, and control stable clones were established from SW620 colon cancer cells derived from a colon cancer lymph node metastasis. Compared with HCT116 cells, relative TIG1A mRNA levels were higher in SW620 cells. However, TIG1B mRNA levels were lower in SW620 cells (Figure 6A). Upon exposure to 5 nM MFP for 24 to 48 h, enhanced GRK5 protein expression was accompanied by induced expression of the TIG1A or TIG1B recombinant protein isoform in the respective stable, but not in control cells (Figure 6B). Growth in TIG1A and TIG1B stable SW620 cells was decreased (compared to growth in control stable cells) by 39 and 39.8%, respectively, upon exposure to 100 nM MFP (Figure 6C). The release of lactate dehydrogenase was determined in stable cells exposed to 5 nM MFP for 1 to 2 days; no increase in cell death was observed (data not shown).

## Discussion

TIG1 is a retinoid-inducible tumour suppressor. Although studies have demonstrated that TIG1B suppresses cell growth and invasion, the function of TIG1A has not been reported. In this study, both TIG1A and TIG1B were downregulated in colon cancer cell lines, and their expression suppressed the growth of colon cancer cells. Various genes involved in cell signaling pathways associated with the regulation of cellular growth, differentiation, and apoptosis were differentially expressed followed the induction of TIG1A or TIG1B expression in HCT116 cells, while expression of GRK5, a protein that desensitises GPCR signaling, was upregulated by both TIG1 isoforms. GRK5 silencing alleviated TIG1A-mediated growth suppression by 60.5%. The extent of growth reversion and inhibition of GRK5 expression was largely similar in TIG1A expressing cells transfected with GRK5 siRNA, suggesting that GRK5 plays a pivotal role in TIG1-mediated growth inhibition of HCT116 colon cancer cells.

Loss of TIG1 expression through promoter DNA hypermethylation occurs in many tumour tissues [5-11], suggesting that the loss of TIG1 provides a survival advantage to tumour cells. This notion is supported by findings from this and previous studies [2,7,10] demonstrating the growth and invasion suppressive activities of TIG1 in prostate, endometrial, nasopharyngeal, and colon cancer cells. The strong growth suppressive activities of TIG1 are also supported by the unsuccessful establishment of TIG1B stable cells from endometrial cancer cells observed previously [10]. Similarly, we have failed to establish constitutive TIG1A and TIG1B stable clones from CC-M2, SW620, and HCT116 colon cancer cells. Therefore, the inducible GeneSwitch system, with reported low basal expression, was used in our study [18,20].

In our study, both TIG1 isoforms exhibited growth suppressive activities in transiently transfected HCT116 cells and TIG1-expressing stable HCT116 and SW620 cells. However, only TIG1B was shown to induce cell death of transiently transfected HCT116 cells. This may explain the greater inhibition of colony formation in TIG1B-transfected HCT116 cells, compared with in TIG1A transfected cells. In inducible stable cells, MFP alone exhibited growth suppressive activity in both HCT116 and SW620 cells. This may be related to the relatively weak (22.2 to 39.8%) TIG1-mediated growth inhibition of stable TIG1A and TIG1B expressing cells.

MFP, a competitive progesterone receptor antagonist, serves as progesterone receptor or estrogen receptor agonist in the absence of progesterone [23] or estrogen [24], respectively. Colon cancer tissues express estrogen and progesterone receptors [25]. Several studies have demonstrated that the estrogen receptor may play a role in the growth inhibition of colon cancer cells *in vitro *[26] and *in vivo *[27]. This may explain the weak growth suppressive activity of MFP on stable control HCT116 and SW620 cells. The weak growth suppressive activity of MFP on colon cancer cells may mask the growth suppressive and/or cell death inducing activities of the TIG1A and TIG1B isoforms on colon cancer cells. This may be related to the lack of cell death induction in both stable TIG1 cells established from HCT116 and SW620 cells followed by TIG1A or TIG1B expression upon MFP exposure. We also examined cell cycle progression in stable HCT116 cells and found that TIG1A, but not TIG1B, significantly decreased the progression of cells from the G1 phase to the S phase in MFP (5 nM)-treated, serum-deprived stable HCT116 cells re-stimulated with complete medium for 12 h (Additional file 6). Therefore, TIG1 isoforms may induce growth suppression of colon cancer cells through multiple mechanisms.

GRK5 is a serine/threonine kinase and is ubiquitously expressed in most tissues [28], suggesting the physiological significance of this protein. GRK5 desensitises the activation of the GPCR signaling pathway by inducing the phosphorylation of agonist-bound GPCR, which in turn leads to uncoupling of the receptor from the G protein and subsequent inhibition of signaling [19]. In addition to inhibiting GPCR-mediated physiological processes, the activity of GRK5 is also mediated through non-GPCR pathways. For example, GRK5 directly phosphorylates the PPPSP motifs on the single transmembrane low density lipoprotein receptor-related protein 6 (LRP6) and stimulates Wnt/LRP6 signaling [29]. GRK5 is also expressed in the nucleus and functions as a kinase for p53 [30] and histone deacetylase [31]. Further, GRK5-mediated phosphorylation increases histone deacetylase activity [32] and promotes p53 degradation [30]. Finally, GRK5 binds IκBα, inducing nuclear accumulation and leading to suppression of NFκB-mediated transactivation and cell growth *in vitro *and *in vivo *[33,34].

In the present study, we observed partial, but significant, reversion of TIG1-mediated growth suppression in TIG1A-expressing HCT116 cells after transfection with TIG1 and GRK5 siRNA. The partial reversion of TIG1-mediated growth inhibition may due to incomplete knockdown of TIG1A and GRK5 protein expression (see Figure 5A). The fact that knockdown of GRK5 expression using GRK5 siRNAs significantly alleviated TIG1A-mediated growth inhibition suggests that GRK5 plays an important role in regulating TIG1A-mediated growth in HCT116 cells. GRK5 participates in multiple signaling pathways at the plasma membrane and nucleus (as described above), which may explain the slightly higher, although not statistically significant, reversion of TIG1A-mediated growth suppression in TIG1A expressing cells using GRK5 siRNA compared with the cells transfected with TIG1A siRNA. Elevated PGE2 levels, which lead to enhanced GPCR signaling, play an important role in colorectal carcinogenesis [35]. Whether the effects of GRK5 on TIG1A-mediated cellular changes are related to inhibition of GPCR, non-GPCR, or both pathways merits further investigation.

In TIG1A-expressing cells, genes participating in p53 (seven in absentia homolog 1 [SIAH1]), Wnt (protein phosphatase 3 [PPP3CA], TCF7, AT rich interactive domain 1A [ARID1A], SIAH1, and Nuclear factor of activated T-cells, cytoplasmic 1 [NFATC1]), chemokine and cytokine (NFATC1, Rho-associated, coiled-coil containing protein kinase 2 [ROCK2], signal transducer and activator of transcription 2 [STAT2], and forkhead box D1 [FOXD1]), and GPCR (guanine nucleotide binding protein, alpha stimulating [GNAS]) signaling pathways were differentially upregulated. In addition, genes participating in angiogenesis (fibroblast growth factor receptors 2 and 3 [FGFR2 and FGFR3], protein tyrosine phosphatase, non-receptor type 6 [PTPN6], STAT2, and platelet-derived growth factor C [PDGFC]) and fibroblast growth factor signaling (FGFR2, PPP2R2C and FGFR3) were specifically upregulated. Furthermore, most signal pathways involving genes differentially regulated by TIG1B were similarly expressed in TIG1A expressing cells. Although TIG1 proteins belong to the latexin protein family, only the TIG1A isoform contains the complete latexin domain that spans amino acids 51 to 276. The TIG1B isoform contains only the N-terminal portion of the latexin domain. A difference in the carboxyl terminus between the 2 TIG1 isoforms may explain why TIG1A differentially regulated more genes associated with cellular growth, apoptosis, and protein transportation and secretion than TIG1B. Despite the structural difference at the carboxyl terminus, both TIG1 isoforms suppressed cell growth. Thus, the N-terminal 224 amino acid fragment of TIG1A may underlie growth suppressive activity.

The present study also demonstrated that TIG1A but not TIG1B delayed progression from the G1 phase to the S phase in MFP-treated, serum-starved stable HCT116 cells after re-exposure to serum containing medium for 12 h (Additional file 6). This result suggests that the growth-related activities of the TIG1 protein may also reside in the carboxyl terminus region of the TIG1A protein, which is supported by the differential expression of a larger number of genes in TIG1A-expressing stable cells compared with TIG1B-expressing cells. Further analysis of the correlation between protein structure and biological activities of TIG1 will be important to elucidate the function of TIG1 proteins.

## Conclusion

In conclusion, we have demonstrated decreased expression of both TIG1 isoforms in colon cancer cell lines compared with expression in normal tissue. In addition, both TIG1 isoforms similarly suppressed colon cancer cell growth. Our findings suggest that TIG1-mediated growth suppression of colon cancer cells is mediated, at least in part, through GRK5. Further analysis of the genes differentially regulated by TIG1A and TIG1B expression will expand our understanding of the molecular mechanism of TIG1-mediated growth suppression in cancer cells.

## Competing interests

The authors declare that they have no competing interests.

## Authors' contributions

CCW initiated the study, performed experiments analysing the expression and activities of TIG1 isoforms using transient transfection, and contributed to the experimental design. FMT performed the majority of the experiments, contributed to the experimental design, and drafted the manuscript. RYS and CHW contributed to experimental design and data discussion. YMT established inducible HCT116 stable cell lines. SYJ designed and supervised the experiments, assisted in the writing of and proofed the manuscript. All authors read and approved the final draft of the manuscript.

## Acknowledgements

The study was supported in part by grants from the National Science Council (NSC 96-2320-B-303-003-MY3 and NSC 96-2314-B-303-010), the Buddhist Tzu Chi General Hospital, Taipei Branch (TCRD-TPE-96-C1-1), the National Defense Medical Center (DOH97-28-07-01), and the Tri-Service General Hospital (TSGH-C98-13-S04-012) Taipei, Taiwan. The authors thank the Core Laboratory of the Buddhist Tzu Chi General Hospital for facility support and Ms. Su-Ching Lin for technical assistance.

## Pre-publication history

The pre-publication history for this paper can be accessed here:

http://www.biomedcentral.com/1471-2407/11/175/prepub

## Supplementary Material

Additional file 1 **Effect of TIG1 isoform expression on HCT116 cell death**. HCT116 cells were plated in triplicate in 6-well plates overnight and then transfected with constitutive TIG1A, TIG1B or RIG1 expression vector or control vector. Supernatants were collected 1 to 3 days after transfection and lactate dehydrogenase activity was measured using a cytotoxicity detection kit (Roche, Nonnenwald, Germany). Lactate dehydrogenase activity was normalised to that of control-transfected cells. Cells transfected with the RIG1 expression vector, a retinoid-inducible proapoptotic protein, served as the positive control. Student's *t *test: *. *P *< 0.05; **, *P *< 0.01; ***, *P *< 0.001. Click here for file

Additional file 2 **Genes differentially regulated by TIG1A or TIG1B in HCT116 cells**. Control (Ctrl), TIG1A, and TIG1B stable cells were treated with 5 nM MFP for 24 h. Gene expression profiles were then determined using microarray analysis. After comparing to the gene expression in control cells, genes upregulated or downregulated by greater than two-fold in TIG1A (A) or TIG1B (B) stable cells were subjected to hierarchical clustering. Average expression was defined using GeneSpring^® ^software. The relative scale of upregulation (red) or downregulation (green) of gene expression is shown in the right panels. Click here for file

Additional file 3 **List of genes differentially regulated by the expression of TIG1A in HCT116 cells**. Click here for file

Additional file 4 **List of genes differentially regulated by the expression of TIG1B in HCT116 cells**. Click here for file

Additional file 5 **List of genes differentially regulated by both TIG1A and TIG1B in HCT116 cells**. Click here for file

Additional file 6 **Effects of TIG1A and TIG1B on cell cycle progression in HCT116 cells**. Control, TIG1A, and TIG1B stable cells were plated overnight and then incubated with 5 nM MFP containing complete medium for 24 h. Cells were serum starved for 24 h and then stimulated with complete medium for the indicated periods. Cells were harvested, fixed, and then incubated in propidium iodide solution. Cell cycle phase was analysed using Cytometrics FC500 (Beckman Coulter, Fullerton, CA, USA), and cell cycle phase distribution was analysed using the MultiCycle. Click here for file
