# Supplementary figures and images for: G protein-coupled receptor kinase 5 mediates Tazarotene-induced gene 1-induced growth suppression of human colon cancer cells
Source: BMC Cancer. 2011 May 17;11:175. doi: 10.1186/1471-2407-11-175 (PMC3112162; doi:10.1186/1471-2407-11-175)

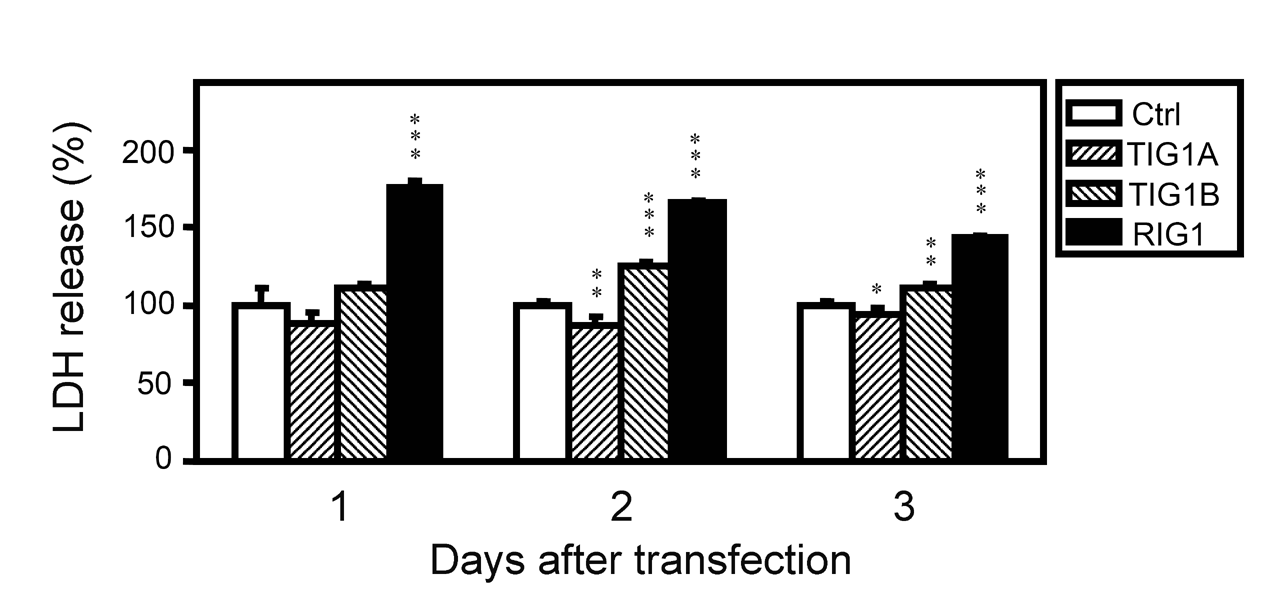

Supplement: Additional file 1 — Effect of TIG1 isoform expression on HCT116 cell death. HCT116 cells were plated in triplicate in 6-well plates overnight and then transfected with constitutive TIG1A, TIG1B or RIG1 expression vector or control vector. Supernatants were collected 1 to 3 days after transfection and lactate dehydrogenase activity was measured using a cytotoxicity detection kit (Roche, Nonnenwald, Germany). Lactate dehydrogenase activity was normalised to that of control-transfected cells. Cells transfected with the RIG1 expression vector, a retinoid-inducible proapoptotic protein, served as the positive control. Student's t test: *. P < 0.05; **, P < 0.01; ***, P < 0.001. [file 1471-2407-11-175-S1.TIFF]

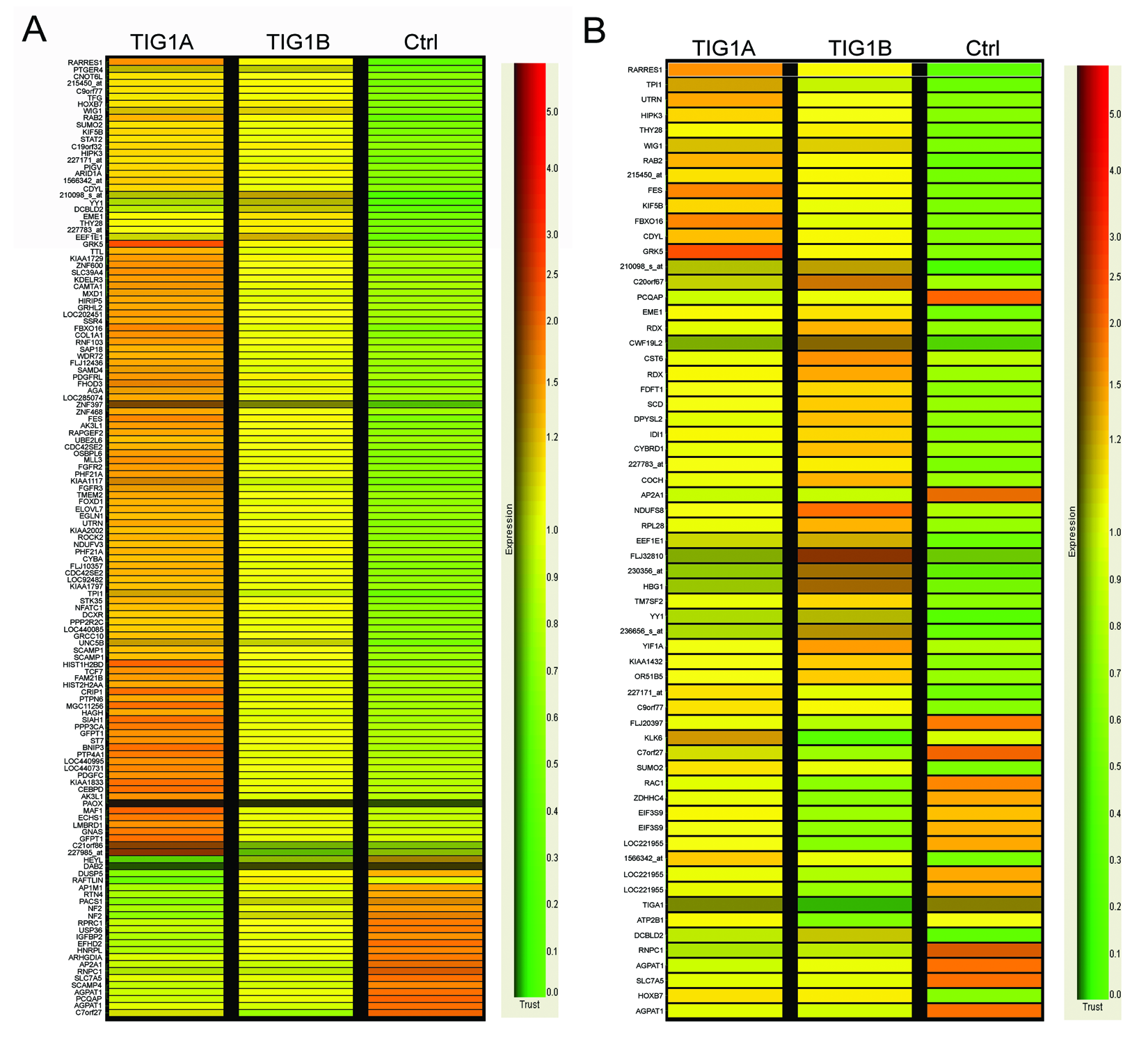

Supplement: Additional file 2 — Genes differentially regulated by TIG1A or TIG1B in HCT116 cells. Control (Ctrl), TIG1A, and TIG1B stable cells were treated with 5 nM MFP for 24 h. Gene expression profiles were then determined using microarray analysis. After comparing to the gene expression in control cells, genes upregulated or downregulated by greater than two-fold in TIG1A (A) or TIG1B (B) stable cells were subjected to hierarchical clustering. Average expression was defined using GeneSpring® software. The relative scale of upregulation (red) or downregulation (green) of gene expression is shown in the right panels. [file 1471-2407-11-175-S2.TIFF]

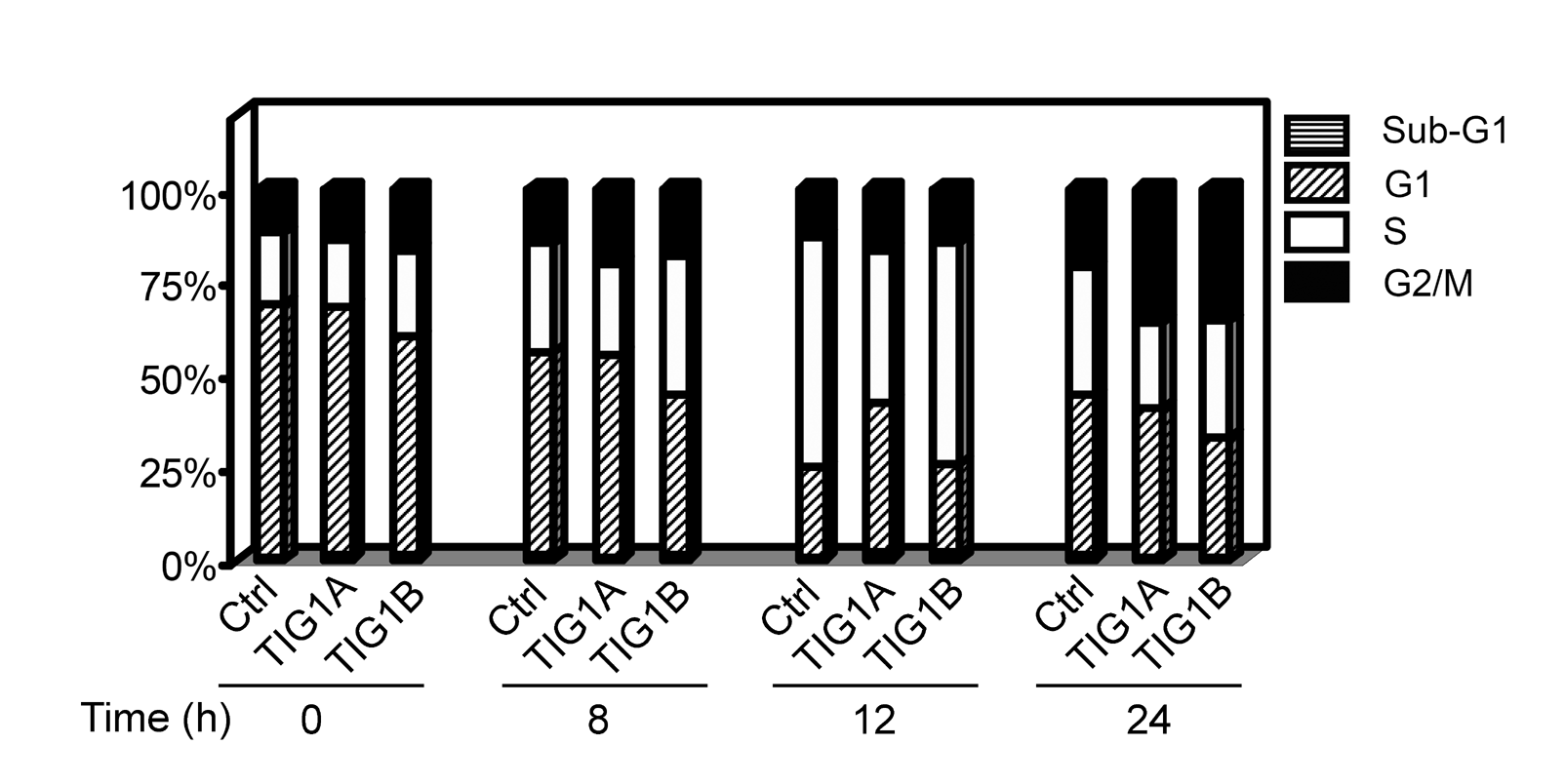

Supplement: Additional file 6 — Effects of TIG1A and TIG1B on cell cycle progression in HCT116 cells. Control, TIG1A, and TIG1B stable cells were plated overnight and then incubated with 5 nM MFP containing complete medium for 24 h. Cells were serum starved for 24 h and then stimulated with complete medium for the indicated periods. Cells were harvested, fixed, and then incubated in propidium iodide solution. Cell cycle phase was analysed using Cytometrics FC500 (Beckman Coulter, Fullerton, CA, USA), and cell cycle phase distribution was analysed using the MultiCycle. [file 1471-2407-11-175-S6.TIFF]
