# Supplementary material for: G protein-coupled receptor kinase 5 mediates Tazarotene-induced gene 1-induced growth suppression of human colon cancer cells
Source: BMC Cancer. 2011 May 17;11:175. doi: 10.1186/1471-2407-11-175 (PMC3112162; doi:10.1186/1471-2407-11-175)
Supplement: Additional file 3 — List of genes differentially regulated by the expression of TIG1A in HCT116 cells. [file 1471-2407-11-175-S3.DOC]

Supplementary Table I. List of genes differentially regulated by the expression of TIG1A in HCT116 cells.

| Gene Name^a^ |  | GenBank/EMBL/DDB accession number | P-value | Fold Change |  | Gene Symbol | Description |
| --- | --- | --- | --- | --- | --- | --- | --- |
| 206392_s_at |  | NM_206963 | <0.01 | 166.10 |  | RARRES1 | retinoic acid receptor responder (tazarotene induced) 1 |
| 221960_s_at |  | NM_002865 | <0.001 | 5.43 |  | RAB2 | RAB2, member RAS oncogene family |
| 204395_s_at |  | NM_005308 | <0.01 | 5.06 |  | GRK5 | G protein-coupled receptor kinase 5 |
| 205418_at |  | NM_002005 | <0.01 | 3.90 |  | FES | feline sarcoma oncogene |
| 210098_s_at |  | AF130102 | <0.01 | 3.87 |  |  | predicted protein of HQ0522 |
| 209911_x_at |  | NM_021063 | <0.01 | 3.86 |  | HIST1H2BD | histone 1, H2bd |
| 228427_at |  | NM_172366 | <0.01 | 3.74 |  | FBXO16 | F-box protein 16 |
| 225342_at |  | Hs.592601 | <0.05 | 3.20 |  | AK3L1 | adenylate kinase 3-like 1 |
| 1566342_at |  |  | <0.01 | 3.13 |  |  | Transcribed locus |
| 1556499_s_at |  | NM_013410 | <0.05 | 3.13 |  | COL1A1 | collagen, type I, alpha 1 |
| 202981_x_at |  | NM_003031 | <0.01 | 3.12 |  | SIAH1 | seven in absentia homolog 1 (Drosophila) |
| 223805_at |  | NM_032523 | <0.01 | 3.11 |  | OSBPL6 | oxysterol binding protein-like 6 |
| 222415_at |  | NM_170606 | <0.01 | 3.11 |  | MLL3 | myeloid/lymphoid or mixed-lineage leukemia 3 |
| 205081_at |  | NM_001311 | <0.05 | 3.10 |  | CRIP1 | cysteine-rich protein 1 (intestinal) |
| 227171_at |  | NM_006430 | <0.01 | 3.10 |  | CCT4 | Chaperonin containing TCP1, subunit 4 (delta) |
| 218358_at |  | CV868772 | <0.05 | 3.08 |  | MGC11256 | hypothetical protein MGC11256 |
| 201848_s_at |  | NM_004052 | <0.01 | 3.01 |  | BNIP3 | BCL2/adenovirus E1B 19kDa interacting protein 3 |
| 203638_s_at |  | NM_000141 | <0.01 | 3.00 |  | FGFR2 | fibroblast growth factor receptor 2 |
| 215450_at |  | NM_003094 | <0.05 | 2.99 |  | SNRPE | Small nuclear ribonucleoprotein polypeptide E |
| 218980_at |  | NM_025135 | <0.05 | 2.96 |  | FHOD3 | formin homology 2 domain containing 3 |
| 225093_at |  | NM_007124 | <0.05 | 2.92 |  | UTRN | utrophin (homologous to dystrophin) |
| 210050_at |  | NM_000365 | <0.05 | 2.84 |  | TPI1 | triosephosphate isomerase 1 |
| 213271_s_at |  | AB029040.2 | <0.01 | 2.77 |  | KIAA1117 | KIAA1117 |
| 222998_at |  | NM_032272 | <0.01 | 2.76 |  | MAF1 | MAF1 homolog (S. cerevisiae) |
| 234464_s_at |  | NM_152463.2 | <0.01 | 2.72 |  | EME1 | essential meiotic endonuclease 1 homolog 1 (S. pombe) |
| 230361_at |  | AB058736 | <0.05 | 2.70 |  | KIAA1833 | hypothetical protein KIAA1833 |
| 203973_s_at |  | NM_005195 | <0.05 | 2.69 |  | CEBPD | CCAAT/enhancer binding protein (C/EBP), delta |
| 203278_s_at |  | NM_001101802 | <0.05 | 2.66 |  | PHF21A | PHD finger protein 21A |
| 200732_s_at |  | NM_003463 | <0.01 | 2.65 |  | PTP4A1 | protein tyrosine phosphatase type IVA, member 1 |
| 58780_s_at |  | NM_018071 | <0.05 | 2.65 |  | FLJ10357 | hypothetical protein FLJ10357 |
| 219215_s_at |  | NM_017767 | <0.01 | 2.63 |  | SLC39A4 | solute carrier family 39 (zinc transporter), member 4 |
| 50400_at |  | NM_152911 | <0.05 | 2.63 |  | PAOX | polyamine oxidase (exo-N4-amino) |
| 225649_s_at |  | NM_080836 | <0.01 | 2.61 |  | STK35 | serine/threonine kinase 35 |
| 229606_at |  | NM_000944 | <0.05 | 2.60 |  | PPP3CA | Protein phosphatase 3, catalytic subunit, alpha isoform, calcineurin A |
| 202762_at |  | NM_004850 | <0.01 | 2.60 |  | ROCK2 | Rho-associated, coiled-coil containing protein kinase 2 |
| 203098_at |  | NM_004824 | <0.01 | 2.54 |  | CDYL | chromodomain protein, Y-like |
| 213879_at |  | NM_006937 | <0.05 | 2.50 |  | SUMO2 | SMT3 suppressor of mif two 3 homolog 2 (yeast) |
| 201991_s_at |  | NM_004521 | <0.05 | 2.49 |  | KIF5B | kinesin family member 5B |
| 224709_s_at |  | NM_020240 | <0.01 | 2.48 |  | CDC42SE2 | CDC42 small effector 2 |
| 202636_at |  | NM_005667 | <0.01 | 2.48 |  | RNF103 | ring finger protein 103 |
| 228125_at |  | NM_001135178 | <0.05 | 2.48 |  | ZNF397 | Zinc finger protein 397 |
| 223711_s_at |  | CV869019 | <0.05 | 2.48 |  | THY28 | thymocyte protein thy28 |
| 203097_s_at |  | NM_014247 | <0.05 | 2.46 |  | RAPGEF2 | Rap guanine nucleotide exchange factor (GEF) 2 |
| 204380_s_at |  | NM_000142 | <0.05 | 2.42 |  | FGFR3 | fibroblast growth factor receptor 3 |
| 228051_at |  | DN989653.1 | <0.05 | 2.42 |  | LOC202451 | hypothetical protein LOC202451 |
| 213907_at |  | NM_004280 | <0.01 | 2.36 |  | EEF1E1 | Eukaryotic translation elongation factor 1 epsilon 1 |
| 230588_s_at |  | NR_026846 | <0.01 | 2.36 |  | LOC285074, LOC388972 | hypothetical protein LOC285074 |
| 213873_at |  | NM_080927 | <0.05 | 2.35 |  | DCBLD2 | discoidin, CUB and LCCL domain containing 2 |
| 206307_s_at |  | NM_004472 | <0.01 | 2.34 |  | FOXD1 | forkhead box D1 |
| 218722_s_at |  | AK022498.1 | <0.01 | 2.32 |  | FLJ12436 | hypothetical protein FLJ12436 |
| 242463_x_at |  | NM_198457.2 | <0.01 | 2.32 |  | ZNF600 | zinc finger protein 600 |
| 215495_s_at |  | CX063669 | <0.05 | 2.31 |  | SAMD4 | sterile alpha motif domain containing 4 |
| 202722_s_at |  | NM_002056.2 | <0.01 | 2.30 |  | GFPT1 | glutamine-fructose-6-phosphate transaminase 1 |
| 213494_s_at |  | NM_003403 | <0.05 | 2.29 |  | YY1 | YY1 transcription factor |
| 237563_s_at |  | XM_933693.3 | <0.01 | 2.28 |  | LOC440731 | LOC440731 |
| 213220_at |  | BC073157 | <0.01 | 2.27 |  | LOC92482 | hypothetical protein LOC92482 |
| 226297_at |  | NM_005734 | <0.01 | 2.27 |  | HIPK3 | Homeodomain interacting protein kinase 3 |
| 227783_at |  | NM_198082 | <0.05 | 2.26 |  | CCDC57 | Coiled-coil domain containing 57 |
| 224719_s_at |  | DR005775 | <0.05 | 2.26 |  | GRCC10 | likely ortholog of mouse gene rich cluster, C10 gene |
| 201135_at |  | NM_004092 | <0.05 | 2.25 |  | ECHS1 | enoyl Coenzyme A hydratase, short chain, 1, mitochondrial |
| 218113_at |  | NM_013390 | <0.05 | 2.23 |  | TMEM2 | transmembrane protein 2 |
| 212929_s_at |  | NM_018232.1 | <0.05 | 2.23 |  | FAM21B FAM21C LOC387680  LOC439973 | family with sequence similarity 21, member B |
| 213268_at |  | NM_015215 | <0.05 | 2.22 |  | CAMTA1 | calmodulin binding transcription activator 1 |
| 227985_at |  |  | <0.01 | 2.22 |  |  | Transcribed locus |
| 211105_s_at |  | NM_172390 | <0.01 | 2.22 |  | NFATC1 | nuclear factor of activated T-cells, cytoplasmic, calcineurin-dependent 1 |
| 207871_s_at |  | NM_018412 | <0.01 | 2.22 |  | ST7 | suppression of tumorigenicity 7 |
| 217973_at |  | NM_016286 | <0.05 | 2.21 |  | DCXR | dicarbonyl/L-xylulose reductase |
| 227551_at |  | DN992915 | <0.01 | 2.21 |  | C9orf77 | chromosome 9 open reading frame 77 |
| 204017_at |  | NM_006855 | <0.05 | 2.21 |  | KDELR3 | KDEL (Lys-Asp-Glu-Leu) endoplasmic reticulum protein retention receptor 3 |
| 226899_at |  | NM_170744 | <0.01 | 2.21 |  | UNC5B | unc-5 homolog B (C. elegans) |
| 222719_s_at |  | NM_016205 | <0.05 | 2.20 |  | PDGFC | platelet derived growth factor C |
| 201004_at |  | NM_006280 | <0.01 | 2.20 |  | SSR4 | signal sequence receptor, delta (translocon-associated protein delta) |
| 205226_at |  | NM_006207 | <0.05 | 2.20 |  | PDGFRL | platelet-derived growth factor receptor-like |
| 227180_at |  | NM_024930 | <0.01 | 2.20 |  | ELOVL7 | ELOVL family member 7, elongation of long chain fatty acids (yeast) |
| 225913_at |  | AB082533.1 | <0.01 | 2.20 |  | KIAA2002 | KIAA2002 protein |
| 235911_at |  | XM_002345728 | <0.01 | 2.19 |  | LOC440995 | hypothetical gene supported by BC034933; BC068085 |
| 212416_at |  | NM_004866 | <0.05 | 2.19 |  | SCAMP1 | secretory carrier membrane protein 1 |
| 204778_x_at |  | NM_004502 | <0.05 | 2.18 |  | HOXB7 | homeo box B7 |
| 226909_at |  | BC067880 | <0.05 | 2.18 |  | KIAA1729 | KIAA1729 protein |
| 205255_x_at |  | NM_003202 | <0.05 | 2.18 |  | TCF7 | transcription factor 7 (T-cell specific, HMG-box) |
| 228010_at |  | NM_020416 | <0.01 | 2.18 |  | PPP2R2C | protein phosphatase 2 (formerly 2A), regulatory subunit B (PR 52), gamma isoform |
| 204897_at |  | NM_000958 | <0.05 | 2.17 |  | PTGER4 | prostaglandin E receptor 4 (subtype EP4) |
| 218917_s_at |  | NM_006015 | <0.05 | 2.17 |  | ARID1A | AT rich interactive domain 1A (SWI- like) |
| 223046_at |  | NM_022051 | <0.05 | 2.17 |  | EGLN1 | egl nine homolog 1 (C. elegans) |
| 214751_at |  | NM_199132 | <0.05 | 2.17 |  | ZNF468 | zinc finger protein ZNF468 |
| 1555609_a_at |  | AY037945 | <0.05 | 2.16 |  | WIG1 | p53 target zinc finger protein |
| 214157_at |  | NM_000516 | <0.05 | 2.16 |  | GNAS | GNAS complex locus |
| 225636_at |  | NM_005419 | <0.01 | 2.16 |  | STAT2 | signal transducer and activator of transcription 2, 113kDa |
| 216064_s_at |  | NM_000027 | <0.05 | 2.15 |  | AGA | aspartylglucosaminidase |
| 218946_at |  | AJ132584 | <0.01 | 2.14 |  | HIRIP5 | HIRA interacting protein 5 |
| 51146_at |  | NM_017837.2 | <0.05 | 2.12 |  | PIGV | phosphatidylinositol glycan, class V |
| 214290_s_at |  | AK312163 | <0.05 | 2.12 |  | HIST2H2AA | histone 2, H2aa |
| 228846_at |  | NM_002357 | <0.05 | 2.12 |  | MXD1 | MAX dimerization protein 1 |
| 228908_s_at |  | AF426264 | <0.05 | 2.10 |  | C21orf86 | Chromosome 21 open reading frame 86 |
| 218191_s_at |  | NM_018368.2 | <0.01 | 2.08 |  | LMBRD1 | LMBR1 domain containing 1 |
| 201649_at |  | NM_004223 | <0.05 | 2.07 |  | UBE2L6 | ubiquitin-conjugating enzyme E2L 6 |
| 218503_at |  | NM_017794 | <0.01 | 2.07 |  | KIAA1797 | KIAA1797 |
| 221871_s_at |  | NM_006070 | <0.05 | 2.06 |  | TFG | TRK-fused gene |
| 219388_at |  | NM_024915 | <0.01 | 2.05 |  | GRHL2 | grainyhead-like 2 (Drosophila) |
| 226616_s_at |  | NM_021075 | <0.05 | 2.05 |  | NDUFV3 | NADH dehydrogenase (ubiquinone) flavoprotein 3, 10kDa |
| 203028_s_at |  | NM_000101 | <0.05 | 2.05 |  | CYBA | cytochrome b-245, alpha polypeptide |
| 208742_s_at |  | NM_005870 | <0.01 | 2.05 |  | SAP18 | sin3-associated polypeptide, 18kDa |
| 224896_s_at |  | NM_153712 | <0.05 | 2.03 |  | TTL | tubulin tyrosine ligase |
| 227174_at |  | NM_182758 | <0.05 | 2.03 |  | WDR72 | WD repeat domain 72 |
| 216384_x_at |  | AF257099.1 | <0.05 | 2.02 |  | LOC440085 | similar to prothymosin alpha |
| 206687_s_at |  | NM_002831 | <0.05 | 2.02 |  | PTPN6 | protein tyrosine phosphatase, non-receptor type 6 |
| 205012_s_at |  | NM_005326 | <0.05 | 2.01 |  | HAGH | hydroxyacylglutathione hydrolase |
| 226597_at |  | NM_138393.1 | <0.05 | 2.00 |  | C19orf32 | chromosome 19 open reading frame 32 |
| 226153_s_at |  | NM_144571 | <0.01 | 2.00 |  | CNOT6L | CCR4-NOT transcription complex, subunit 6-like |
| 226828_s_at |  | NM_014571 | <0.01 | 0.50 |  | HEYL | hairy/enhancer-of-split related with YRPW motif-like |
| 223024_at |  | NM_001130524 | <0.01 | 0.49 |  | AP1M1 | adaptor-related protein complex 1, mu 1 subunit |
| 225437_s_at |  | NM_152743 | <0.01 | 0.49 |  | C7orf27 | chromosome 7 open reading frame 27 |
| 211716_x_at |  | NM_004309 | <0.01 | 0.49 |  | ARHGDIA | Rho GDP dissociation inhibitor (GDI) alpha |
| 234068_s_at |  | NM_014203 | <0.05 | 0.48 |  | AP2A1 | adaptor-related protein complex 2, alpha 1 subunit |
| 212430_at |  | AL928599 | <0.05 | 0.48 |  | RNPC1 | RNA-binding region (RNP1, RRM) containing 1 |
| 224658_x_at |  | NM_018026.2 | <0.01 | 0.47 |  | PACS1 | phosphofurin acidic cluster sorting protein 1 |
| 213244_at |  | NM_079834 | <0.01 | 0.47 |  | SCAMP4 | secretory carrier membrane protein 4 |
| 201280_s_at |  | NM_001343 | <0.01 | 0.47 |  | DAB2 | disabled homolog 2, mitogen-responsive phosphoprotein (Drosophila) |
| 210968_s_at |  | NM_020532 | <0.01 | 0.47 |  | RTN4 | reticulon 4 |
| 201195_s_at |  | NM_003486 | <0.05 | 0.47 |  | SLC7A5 | solute carrier family 7 (cationic amino acid transporter, y+ system), member 5 |
| 32836_at |  | NM_006411 | <0.01 | 0.46 |  | AGPAT1 | 1-acylglycerol-3-phosphate O-acyltransferase 1 (lysophosphatidic acid acyltransferase, alpha) |
| 211091_s_at |  | NM_000268 | <0.01 | 0.45 |  | NF2 | neurofibromin 2 (bilateral acoustic neuroma) |
| 217943_s_at |  | BC003083 | <0.01 | 0.44 |  |  | arginine/proline rich coiled-coil 1 |
| 202718_at |  | NM_000597 | <0.05 | 0.43 |  | IGFBP2 | insulin-like growth factor binding protein 2, 36kDa |
| 227093_at |  | NM_025090 | <0.01 | 0.42 |  | USP36 | Ubiquitin specific peptidase 36 |
| 202072_at |  | AK292115.1 | <0.01 | 0.40 |  | HNRPL | heterogeneous nuclear ribonucleoprotein L |
| 222175_s_at |  | AF328769 | <0.01 | 0.38 |  | PCQAP | PC2 (positive cofactor 2, multiprotein complex) glutamine/Q-rich-associated protein |
| 209457_at |  | NM_004419 | <0.05 | 0.37 |  | DUSP5 | dual specificity phosphatase 5 |
| 217992_s_at |  | NM_024329.5 | <0.05 | 0.37 |  | EFHD2 | EF-hand domain family, member D2 |
| 212646_at |  | DN999266 | <0.01 | 0.26 |  | RAFTLIN | raft-linking protein |

^a^ Name of probe from the Affymetrix HGU-133 Plus 2.0 chip.
