# Supplementary material for: G protein-coupled receptor kinase 5 mediates Tazarotene-induced gene 1-induced growth suppression of human colon cancer cells
Source: BMC Cancer. 2011 May 17;11:175. doi: 10.1186/1471-2407-11-175 (PMC3112162; doi:10.1186/1471-2407-11-175)
Supplement: Additional file 4 — List of genes differentially regulated by the expression of TIG1B in HCT116 cells. [file 1471-2407-11-175-S4.DOC]

Supplementary Table 2. List of genes differentially regulated by the expression of TIG1B in HCT116 cells.

| Gene Name^a^ |  | GenBank/EMBL/DDBJ accession number | P value | Fold Change |  | Gene Symbol | Description |
| --- | --- | --- | --- | --- | --- | --- | --- |
| 206392_s_at |  | NM_206963 | <0.01 | 102.30 |  | RARRES1 | retinoic acid receptor responder (tazarotene induced) 1 |
| 210098_s_at |  | AF130102.1 | <0.01 | 4.43 |  |  | predicted protein of HQ0522 |
| 221960_s_at |  | NM_002865 | <0.01 | 4.12 |  | RAB2 | RAB2, member RAS oncogene family |
| 234464_s_at |  | NM_152463.2 | <0.01 | 2.92 |  | EME1 | essential meiotic endonuclease 1 homolog 1 (S. pombe) |
| 203189_s_at |  | NM_002496 | <0.05 | 2.80 |  | NDUFS8 | NADH dehydrogenase (ubiquinone) Fe-S protein 8 |
| 215450_at |  | NM_003094 | <0.05 | 2.79 |  | SNRPE | Small nuclear ribonucleoprotein polypeptide E |
| 230047_at |  | NM_152432 | <0.05 | 2.76 |  | FLJ32810 | hypothetical protein FLJ32810 |
| 213907_at |  | NM_004280 | <0.01 | 2.70 |  | EEF1E1 | Eukaryotic translation elongation factor 1 epsilon 1 |
| 227171_at |  | NM_006430 | <0.01 | 2.67 |  | CCT4 | Chaperonin containing TCP1, subunit 4 (delta) |
| 213873_at |  | NM_080927 | <0.05 | 2.55 |  | DCBLD2 | discoidin, CUB and LCCL domain containing 2 |
| 223711_s_at |  | CV869019 | <0.05 | 2.55 |  | THY28 | thymocyte protein thy28 |
| 1566342_at |  |  | <0.05 | 2.51 |  |  | Transcribed locus |
| 213494_s_at |  | NM_003403 | <0.05 | 2.49 |  | YY1 | YY1 transcription factor |
| 212398_at |  | NM_002906 | <0.05 | 2.48 |  | RDX | radixin |
| 227783_at |  | NM_198082 | <0.05 | 2.34 |  |  | Coiled-coil domain containing 57 |
| 237040_at |  | NM_152434.2 | <0.01 | 2.34 |  | CWF19L2 | CWF19-like 2, cell cycle control (S. pombe) |
| 230356_at |  |  | <0.05 | 2.33 |  |  | Transcribed locus |
| 205418_at |  | NM_002005 | <0.01 | 2.29 |  | FES | feline sarcoma oncogene |
| 213223_at |  | NM_001136134 | <0.05 | 2.23 |  | RPL28 | ribosomal protein L28 |
| 236656_s_at |  | AF075027.1 | <0.05 | 2.22 |  |  | Full length insert cDNA YI37C01 |
| 208881_x_at |  | NM_004508 | <0.05 | 2.22 |  | IDI1 | isopentenyl-diphosphate delta isomerase 1 |
| 210130_s_at |  | NM_003273 | <0.05 | 2.19 |  | TM7SF2 | transmembrane 7 superfamily member 2 |
| 201991_s_at |  | NM_004521 | <0.05 | 2.18 |  | KIF5B | kinesin family member 5B |
| 213879_at |  | NM_006937 | <0.05 | 2.17 |  | SUMO2 | SMT3 suppressor of mif two 3 homolog 2 (yeast) |
| 200762_at |  | NM_001386 | <0.01 | 2.16 |  | DPYSL2 | dihydropyrimidinase-like 2 |
| 204419_x_at |  | NG_000007.3 | <0.05 | 2.16 |  | HBG1 ; HBG2 | hemoglobin |
| 202418_at |  | NM_020470 | <0.01 | 2.12 |  | YIF1A | Yip1 interacting factor homolog A (S. cerevisiae) |
| 1555609_a_at |  | AY037945 | <0.05 | 2.12 |  | WIG1 | p53 target zinc finger protein |
| 204778_x_at |  | NM_004502 | <0.05 | 2.11 |  | HOXB7 | homeo box B7 |
| 210050_at |  | NM_000365 | <0.05 | 2.10 |  | TPI1 | triosephosphate isomerase 1 |
| 204395_s_at |  | NM_005308 | <0.05 | 2.09 |  | GRK5 | G protein-coupled receptor kinase 5 |
| 227551_at |  | DN992915 | <0.01 | 2.07 |  | C9orf77 | chromosome 9 open reading frame 77 |
| 206595_at |  | NM_001323 | <0.05 | 2.07 |  | CST6 | cystatin E/M |
| 211162_x_at |  | NM_005063 | <0.01 | 2.06 |  | SCD | stearoyl-CoA desaturase (delta-9-desaturase) |
| 203098_at |  | NM_004824 | <0.01 | 2.06 |  | CDYL | chromodomain protein, Y-like |
| 1554242_a_at |  | NM_001135058 | <0.05 | 2.04 |  | COCH | coagulation factor C homolog, cochlin (Limulus polyphemus) |
| 225093_at |  | NM_007124 | <0.05 | 2.03 |  | UTRN | utrophin (homologous to dystrophin) |
| 228427_at |  | NM_172366 | <0.05 | 2.03 |  | FBXO16 | F-box protein 16 |
| 222044_at |  | EU832741 | <0.05 | 2.02 |  | C20orf67 | chromosome 20 open reading frame 67 |
| 226297_at |  | NM_005734 | <0.05 | 2.01 |  | HIPK3 | Homeodomain interacting protein kinase 3 |
| 1570516_s_at |  | NM_001005567.1 | <0.05 | 2.01 |  | OR51B5 | olfactory receptor, family 51, subfamily B, member 5 |
| 217889_s_at |  | NM_024843 | <0.05 | 2.01 |  | CYBRD1 | cytochrome b reductase 1 |
| 208688_x_at |  | DN995459 | <0.05 | 0.50 |  | EIF3S9 | eukaryotic translation initiation factor 3, subunit 9 eta |
| 212430_at |  | AL928599 | <0.05 | 0.49 |  | RNPC1 | RNA-binding region (RNP1, RRM) containing 1 |
| 215535_s_at |  | NM_006411 | <0.05 | 0.49 |  | AGPAT1 | 1-acylglycerol-3-phosphate O-acyltransferase 1 |
| 234068_s_at |  | NM_014203 | <0.01 | 0.49 |  | AP2A1 | adaptor-related protein complex 2, alpha 1 subunit |
| 225832_s_at |  | DR004690 | <0.05 | 0.48 |  | LOC221955 | KCCR13L |
| 209281_s_at |  | NM_001001323 | <0.05 | 0.48 |  | ATP2B1 | ATPase, Ca++ transporting, plasma membrane 1 |
| 201195_s_at |  | NM_003486 | <0.05 | 0.47 |  | SLC7A5 | solute carrier family 7 (cationic amino acid transporter, y+ system), member 5 |
| 222175_s_at |  | AF328769 | <0.01 | 0.44 |  | PCQAP | PC2 (positive cofactor 2, multiprotein complex) glutamine/Q-rich-associated protein |
| 218460_at |  | AK000404.1 | <0.01 | 0.41 |  | FLJ20397 | hypothetical protein FLJ20397 |
| 228256_s_at |  | AB048207.1 | <0.01 | 0.38 |  | C5orf26 | TIGA1 |
| 220261_s_at |  | NM_001134387 | <0.01 | 0.37 |  | ZDHHC4 | zinc finger, DHHC-type containing 4 |
| 204733_at |  | AK314897 | <0.01 | 0.36 |  | KLK6 | kallikrein 6 (neurosin, zyme) |
| 225437_s_at |  | NM_152743 | <0.05 | 0.36 |  | C7orf27 | chromosome 7 open reading frame 27 |
| 1567458_s_at |  | NM_006908 | <0.01 | 0.31 |  | RAC1 | small GTP binding protein Rac1 |

^a^ Name of probe from the Affymetrix HGU-133 Plus 2.0 chip.
