# Supplementary material for: G protein-coupled receptor kinase 5 mediates Tazarotene-induced gene 1-induced growth suppression of human colon cancer cells
Source: BMC Cancer. 2011 May 17;11:175. doi: 10.1186/1471-2407-11-175 (PMC3112162; doi:10.1186/1471-2407-11-175)
Supplement: Additional file 5 — List of genes differentially regulated by both TIG1A and TIG1B in HCT116 cells. [file 1471-2407-11-175-S5.DOC]

Supplementary Table 3. List of genes differentially regulated by both TIG1A and TIG1B in HCT116 cells.

| Gene Name^a^ |  | GenBank/EMBL/DDBJ accession number | Fold Change | |  | Gene Symbol | Description |
| --- | --- | --- | --- | --- | --- | --- | --- |
|  |  |  | TIG1A | TIG1B |  |  |  |
| 206392_s_at |  | NM_206963 | 166.1 | 102.3 |  | RARRES1 | retinoic acid receptor responder (tazarotene induced) 1 |
| 210098_s_at |  | AF130102.1 | 3.9 | 4.4 |  |  | predicted protein of HQ0522 |
| 221960_s_at |  | NM_002865 | 5.4 | 4.1 |  | RAB2 | RAB2, member RAS oncogene family |
| 234464_s_at |  | NM_152463.2 | 2.7 | 2.9 |  | EME1 | essential meiotic endonuclease 1 homolog 1 (S. pombe) |
| 215450_at |  | NM_003094 | 3.0 | 2.8 |  | SNRPE | Small nuclear ribonucleoprotein polypeptide E |
| 213907_at |  | NM_004280 | 2.4 | 2.7 |  | EEF1E1 | Eukaryotic translation elongation factor 1 epsilon 1 |
| 227171_at |  | NM_006430 | 3.1 | 2.7 |  | CCT4 | Chaperonin containing TCP1, subunit 4 (delta) |
| 213873_at |  | NM_080927 | 2.4 | 2.6 |  | DCBLD2 | discoidin, CUB and LCCL domain containing 2 |
| 223711_s_at |  | CV869019 | 2.5 | 2.5 |  | THY28 | thymocyte protein thy28 |
| 1566342_at |  |  | 3.1 | 2.5 |  |  | Transcribed locus |
| 213494_s_at |  | NM_003403 | 2.3 | 2.5 |  | YY1 | YY1 transcription factor |
| 227783_at |  | NM_198082 | 2.3 | 2.3 |  |  | Coiled-coil domain containing 57 |
| 205418_at |  | NM_002005 | 3.9 | 2.3 |  | FES | feline sarcoma oncogene |
| 201991_s_at |  | NM_004521 | 2.5 | 2.2 |  | KIF5B | kinesin family member 5B |
| 213879_at |  | NM_006937 | 2.5 | 2.2 |  | SUMO2 | SMT3 suppressor of mif two 3 homolog 2 (yeast) |
| 1555609_a_at |  | AY037945 | 2.2 | 2.1 |  | WIG1 | p53 target zinc finger protein |
| 204778_x_at |  | NM_004502 | 2.2 | 2.1 |  | HOXB7 | homeo box B7 |
| 210050_at |  | NM_000365 | 2.8 | 2.1 |  | TPI1 | triosephosphate isomerase 1 |
| 204395_s_at |  | NM_005308 | 5.1 | 2.1 |  | GRK5 | G protein-coupled receptor kinase 5 |
| 227551_at |  | DN992915 | 2.2 | 2.1 |  | C9orf77 | chromosome 9 open reading frame 77 |
| 203098_at |  | NM_004824 | 2.5 | 2.1 |  | CDYL | chromodomain protein, Y-like |
| 225093_at |  | NM_007124 | 2.9 | 2.0 |  | UTRN | utrophin (homologous to dystrophin) |
| 228427_at |  | NM_172366 | 3.7 | 2.0 |  | FBXO16 | F-box protein 16 |
| 226297_at |  | NM_005734 | 2.3 | 2.0 |  | HIPK3 | Homeodomain interacting protein kinase 3 |
| 212430_at |  | AL928599 | 0.5 | 0.5 |  | RNPC1 | RNA-binding region (RNP1, RRM) containing 1 |
| 215535_s_at |  | NM_006411 | 0.5 | 0.5 |  | AGPAT1 | 1-acylglycerol-3-phosphate O-acyltransferase 1 |
| 234068_s_at |  | NM_014203 | 0.5 | 0.5 |  | AP2A1 | adaptor-related protein complex 2, alpha 1 subunit |
| 201195_s_at |  | NM_003486 | 0.5 | 0.5 |  | SLC7A5 | solute carrier family 7 (cationic amino acid transporter, y+ system), member 5 |
| 222175_s_at |  | AF328769 | 0.4 | 0.4 |  | PCQAP | PC2 (positive cofactor 2, multiprotein complex) glutamine/Q-rich-associated protein |
| 225437_s_at |  | NM_152743 | 0.5 | 0.4 |  | C7orf27 | chromosome 7 open reading frame 27 |

^a^ Name of probe from the Affymetrix HGU-133 Plus 2.0 chip.
